# Optimization of citrus fiber‐enriched vegan cream cheese alternative and its influence on chemical, physical, and sensory properties

**DOI:** 10.1002/fsn3.4220

**Published:** 2024-05-27

**Authors:** Basak Gurbuz, Merve Cayir, Esra Akdeniz, Saniye Akyıl Öztürk, Safa Karaman, Atefeh Karimidastjerd, Omer Said Toker, İbrahim Palabıyık, Nevzat Konar

**Affiliations:** ^1^ Department of Food Engineering, Chemical and Metallurgical Engineering Faculty Yildiz Technical University Istanbul Turkey; ^2^ Department of Fisheries Technology Engineering, Surmene Faculty of Marine Sciences Karadeniz Technical University Trabzon Turkey; ^3^ Azelis Application and Training Center Istanbul Turkey; ^4^ Department of Food Engineering, Engineering Faculty Niğde Ömer Halisdemir University Nigde Turkey; ^5^ Department of Food Engineering, Agricultural Faculty Tekirdag Namik Kemal University Tekirdag Turkey; ^6^ Department of Dairy Technology, Agriculture Faculty Ankara University Ankara Turkey

**Keywords:** citrus fiber, cream cheese, non‐dairy products, vegan

## Abstract

Dairy product alternatives have increased in recent years as a result of medical prescriptions or personal preferences. The main purpose of the present study was to optimize vegan‐based cream cheese formulation added with citrus fiber considering the textural and physicochemical properties of the samples. The physicochemical (pH value, water activity, and color), texture, microstructure, and sensory properties of manufactured vegan‐based cream cheese were characterized and compared to those of a commercial one. Three optimized products were produced, according to the textural properties. The addition of citrus fiber did not affect the pH and water activity values of the cheese samples significantly. Although citrus fiber had an effect on the color values of the samples, a significant difference in the sensory scores was not recorded by the panelists. The sample having 1.21% citrus fiber (A) showed a hardness value similar to that of control sample and it received high sensory appreciation. The sample added with 1.41% citrus fiber (B) was scored high by the panelists, with no significant difference compared to commercial cream cheese, even though it showed high hardness. According to the results of the current research, vegan‐based cream cheese can be produced as a promising food as a new alternative to milk and dairy products.

## Graphical abstract

The present study is carried out to optimize a vegan‐based cream cheese product using pea protein and citrus fiber using mixture design approach. In the first stage, physicochemical and textural characteristics of the produced cheese samples were determined and the best receipt was created based on the textural analysis and in the second stage, three different cheese samples having different citrus fiber and fat levels were prepared and compared to those of a commercial vegan cream cheese sample.

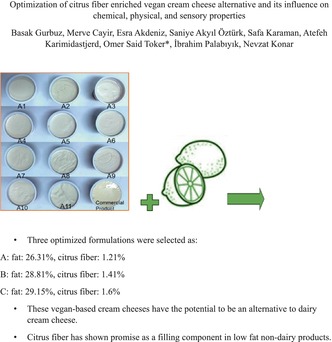

## 1 INTRODUCTION

Given to considerations such as a vegan lifestyle and health concerns, a rising number of individuals are choosing to eat dairy‐free products (Udayarajan et al., 2022). One of the products that can be produced for the vegan consumers is cream cheese (Pombo, 2021). Although the market share of cream cheese is widespread, it cannot be consumed by this special consumer group. Therefore, there is a need for non‐dairy products containing plant‐based protein. Plant‐based fermented foods and beverages have gotten a lot of attention in recent years because of their health benefits and ability to improve food production sustainability (Yang et al., 2021). In addition, veganism has become more popular at present, as has the consumption of dairy products, owing to the rise of hypercholesteremic, milk‐allergic, and lactose‐intolerant people (Angélica Andrade Lopes et al., 2020). On the other hand, consumer demand for milk substitutes is driven by widespread lactose intolerance and cow's milk allergies, as well as ethical concerns and a growing awareness of the need for a more sustainable food system. Due to these consumer groups, there is a need for a variety of vegan products in the market. The number of people who prefer vegetarian and vegan diets is known to be over 600 million globally. When the number of individuals seeking to quit consumption of animal items is added to the number of people not following a strict vegan diet and avoiding solely animal items, it is known that there are more than one billion customers and a substantial market share (Şimşek et al., 2020). Among dairy products, the plant‐based cheese formula is worth investigating (Zhang et al., 2021; Zhang, Jiang, et al., 2024; Zhang, Ling, et al., 2024). Cream cheese is one of the most popular food items commonly consumed around the world. It is a fresh dairy product having a growing economic importance in the food sector, with a global cheese market size of $8.3 billion US dollars expected by 2026. Acid‐coagulated fresh cheese product has a moderate cream buttery flavor and a slight dairy sour taste, with no bitterness, a creamy texture, and a consistency that varies from brittle to spreadable and a shining appearance. Its adaptability allows for a wide range of uses in the food industry. The cheese provides an appropriate matrix for adding flavors, fibers, herbs, condiments, air, or pre‐ and probiotics for example (Pombo, 2021). Cream cheese is semisoft, mildly acidic, has a smooth texture, and has a high dry fat content. After acidification and blending heated cream with milk, rennet was added, followed by whey separation. The curd is cooked and then additives, such as salts or hydrocolloids, can be added (Surber et al., 2021). It is possible to find various cream cheese products with different flavors in the markets. On the other hand, making plant‐based cream cheese and improving its properties could increase its appeal to vegan consumers. A shift toward a more sustainable diet necessitates decreased reliance on animal‐based proteins, prompting the agri‐food industry to seek out novel and alternative protein sources. Plant‐protein‐based dairy and meat substitutes can supply the same amount at a much cheaper cost while decreasing forest devastation and greenhouse gas emissions (Kumar et al., 2022). Many plant‐based protein sources have been reported to create plant‐based milk substitutes and dairy alternatives (Levy et al., 2022). Proteins with excellent technofunctional characteristics, such as lentil protein, pea protein, faba bean protein, and soy protein concentrates, make them good candidates to be used as an emulsifier or a stabilizer (Shi et al., 2020).

Pea protein is a high‐nutrition allergen‐free plant protein composed mostly of 70%–80% globulins and 10%–20% albumins (Li et al., 2022). Due to its minimal allergenicity, non‐transgenic status, high nutritional value and availability, and also being produced from a sustainable crop, pea protein has drawn a lot of attention as a viable option for traditional protein components (animal proteins and soy protein). Pea protein is widely used in vegan products due to its nutritional and technological properties (Boukid et al., 2021). The composition and structure of semisolid meals, which unlike solid foods alter throughout oral processing and require less mastication and faster transport from mouth to the oropharynx, are primarily responsible for hedonic and other sensory properties as well as texture perception (de Wijk et al., 2011). Citrus fibers are utilized as a fat substitute, thickener, and water retention agent in the food formulation (Lundberg et al., 2014). When passing through the stomach, they have been claimed to absorb harmful compounds. In addition, since the citrus fiber does not have an E‐code, consumer perception is not adversely affected (Su et al., 2019). Also, the sensory terminology for fat textural features is highly specific, and the general acceptability of buying reduced‐fat food is mostly dependent on texture attributes perceived directly by the senses (Ningtyas et al., 2019). Fat mimetics can be categorized as carbohydrate‐, protein‐, and fat‐based to produce low‐fat dairy products. Fat‐reduced dairy products are formulated and low‐fat Mozzarella cheese was prepared by applying whey protein‐based fat replacers (Zhang et al., 2021). However, no research has yet been performed on how the texture perception of reduced‐fat vegan‐based cream cheese changes throughout oral ingestion. Moreover, there was no study on the production of vegan cheese with citrus fiber. Previously, citrus fiber as fat replacer and water retention agent has been utilized in food products, such as frankfurter, confectionery, and ice cream (Caggia et al., 2020; Crizel et al., 2014; de Moraes Crizel et al., 2013; Song et al., 2016).

The aim of the present study is to optimize a vegan‐based cream cheese product using pea protein and citrus fiber using mixture design approach. In the first stage, physicochemical and textural characteristics of the produced cheese samples were determined and an ideal receipt was created based on the textural analysis. In the second stage, three different cheese samples having different citrus fiber and fat levels were prepared and their physicochemical, textural, structure, and sensory properties compared to those of commercial vegan cream cheese sample.

## 2 MATERIALS AND METHODS

### 2.1 Materials

Ingredients in the production of vegan‐based cream cheeses including Nutrava Citrus Fiber Peak (fiber 80%) (CP Kelco, Brazil), pea powder (protein 81.7%, carbohydrate 1%, and fiber 7%) (Alfasol, Istanbul, Türkiye), lactic acid 80% (Alfasol, Istanbul, Türkiye), potassium sorbate (Nantong, China), gellan gum (CP Kelco, USA), cheese flavor (Mane, French), coconut oil (Elvan's Food, Istanbul, Türkiye), sugar and salt (Local market, Istanbul, Türkiye) were used in the present study and yeast extract purchased from DSM. Also, commercial cream and vegan‐based cream cheeses were procured from Pınar Food (Izmir, Türkiye) and Bel Karper Food (Tekirdag, Türkiye), respectively.

### 2.2 Production of vegan‐based cream cheese samples

The experimental design was constructed using Design‐Expert software 12 (Minneapolis, MN, USA) and applied to evaluate the effects of fat (A) and citrus fiber (B) on the texture characteristics of the vegan‐based cream cheese. The levels of two factors, citrus fiber and fat levels based on optimal mixture design and experimental design points in terms of coded as 11 combinations, are presented in Table 1. In the formulations, citrus fiber and fat were constrained to range from 1% to 2%, and 15% to 30%, respectively, based on commercial formulation of cream cheese.

**TABLE 1:** Experimental design and mass fraction of three components in vegan‐based cream cheese formulation and its response (hardness) according to simplex lattice mixture design.

Sample	Fat (%)	Citrus fiber (%)	Water (%)	Hardness (g)
1	15	1	78.6	23.8 ± 1.6
2	15	1.5	78.1	8.8 ± 0.5
3	15	2	77.6	25.0 ± 1.6
4	22.5	1	71.1	40.0 ± 0.5
5	22.5	1.5	70.6	18.7 ± 1.5
6	22.5	1.5	70.6	51.0 ± 3.2
7	22.5	1.5	70.6	44.5 ± 1.7
8	22.5	2	70.1	18.1 ± 0.7
9	30	1	63.6	128.5 ± 4.7
10	30	1.5	63	109.4 ± 5.5
11	30	2	62.6	66.7 ± 3.1

**FIGURE 1:**
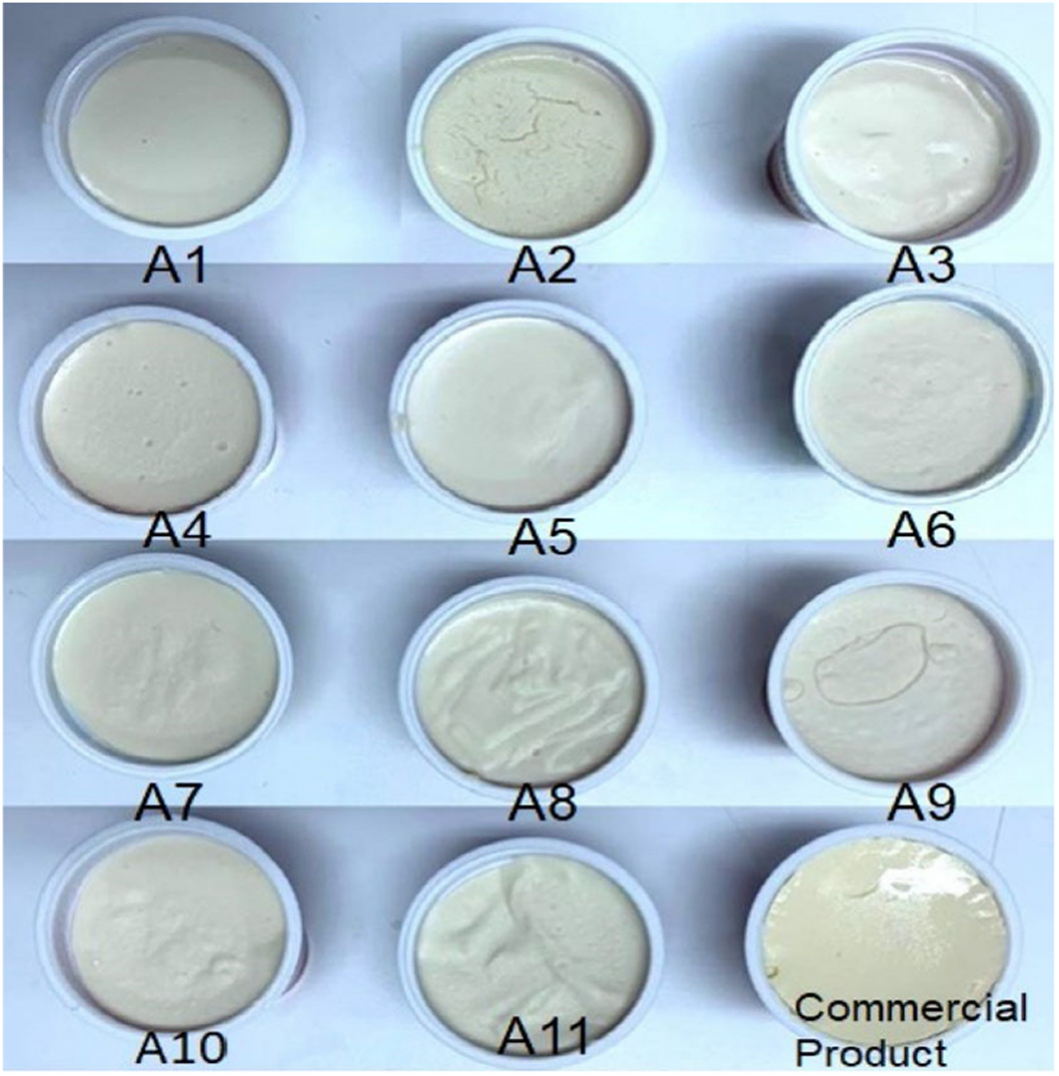
Formulated samples and commercial cream chesses.

The vegan‐based cream cheese samples were produced using coconut oil in Thermomix® at 7500 rpm (revolutions per minute). In order to provide the desired texture in the vegan‐based cream cheese formulas, the coconut oil was utilized, prior to the crystallization of coconut oil that was used in cheese production, also (Masiá et al., 2022). First, the citrus fiber and pea protein were mixed in water for 10 min. After the citrus fiber and pea protein were suspended in water, coconut oil was added and mixed at the same rpm for 5 min (Table 1). The results from ANOVA for hardness of samples, the significance of the overall model, R2, and p‐values of independent factors (A‐fat and B‐citrus) were given in Table 2. Other fixed materials given in Table 3 were added and mixed for 5 min. For the setting of pH values of the mixtures between 4.8 and 5.0, lactic acid (~0.2%) was added, heat treatment was applied at 85°C for 5 min for the samples, and then the samples were filled into commercial cream cheese containers at 50°C. The produced cheese samples were kept at +4°C until analyses. Formulated 11 different samples and commercial vegan‐based cream cheeses are presented in Figure 1.

**TABLE 2:** Analysis of variance (ANOVA) for hardness values of formulated vegan‐based cream cheeses with citrus fiber.

Source	Sum of squares	*df*	Mean square	*F* value	*p*‐Value, Prop > *F*	
Model	14,131.63	4	3532.91	22.41	.0009	Significant*
A‐Fat	10,172.78	1	10,172.78	64.54	.0002	
B‐Citrus	11.6421	1	1164.21	7.39	.0348	
AB	990.74	1	990.74	6.29	.0461	
A^2^	1803.90	1	1803.90	11.44	.0148	
Residual	945.79	6	157.63			
Lack of fit	363.25	4	90.81	0.31	.8525	Not significant
Pure error	582.54	2	291.27			
Cor Total	15,077.41	10				
*R* ^2^	.9373					
Adj *R* ^2^	.8955					
Pred *R* ^2^	.8024					

* Level of significance is *p* < .05.

**TABLE 3:** Ingredient ratios used in vegan‐based cream cheeses.

Ingredient (g/100 g)	Sample A	Sample B	Sample C
Fat	26.32	28.18	29.15
Citrus fiber	1.21	1.41	1.60
Water	67.07	65.01	63.85
Pea powder	3	3	3
Sugar	1.15	1.15	1.15
Lactic acid	0.2	0.2	0.2
Salt	0.8	0.8	0.8
Potassium sorbate	0.1	0.1	0.1
Gellan gum	0.1	0.1	0.1
Yeast extract	0.125	0.125	0.125
Cheese flavor	0.125	0.125	0.125

As textural property is the main critical property at the first step of formulation of vegan‐based cream cheese, three optimum vegan‐based cheese samples were chosen based on the texture analysis results. Following all characteristic analyses were performed on these three selected samples. The general recipes for the cheese samples are shown in Table 3.

### 2.3 Physicochemical analysis

The vegan‐based cream cheese samples were subjected to some physicochemical analyses, such as color, pH, and water activity. A colorimeter (Chroma Meter CR‐400, Konica Minolta, Japan) was used to determine the color parameters of the vegan‐based cream cheeses. L* (whiteness/darkness), a* (redness/greenness), and b* (yellowness/blueness) color parameters of samples were recorded (Karimidastjerd et al., 2024; Perina et al., 2015). Analysis was performed with five replications. The water activity values (*a*_*w*_) of samples were measured by a LabMaster *a*_*w*_ (Novasina, Switzerland) meter by placing the samples in the cup of device. Following the production of samples, pH values were determined using a pH meter (Hanna, USA) after calibration.

### 2.4 Texture analysis

The texture analysis of vegan‐based cream cheese samples was conducted using a texture analyzer (TA.HDPlus, Stable Micro Systems Ltd., Godalming, UK) equipped with a 5 kg load cell. The firmness values of the samples were determined by a modified method (Ong et al., 2020). A cylindrical probe with a diameter of 5 mm was used to measure the firmness of vegan‐based cream cheese samples at a distance of 30 mm and a test speed of 2 mm/s. For all samples, the analysis was carried out with five repetitions.

### 2.5 Microstructure

The microstructure of vegan‐based cream cheese samples was monitored by a polarized light microscope (PLM, Zeiss Axio Imager A2, Carl Zeiss, Germany). Images of the samples were recorded using a 40× objective lens after the samples were placed on the microscope slide that was covered with a coverslip.

### 2.6 Sensory analysis

Sensory characteristics of the vegan‐based cream cheese samples were determined by 30 panel members consisted of graduate students from the Food Engineering Department at Yıldız Technical University. The sensory attributes, such as color, smell, taste, odor, appearance, spreadability, consistency in the mouth, mouthfeel, and general appreciation, were evaluated using hedonic scale (5: the best, 1: the worst score) (Lawless & Heymann, 2010).

### 2.7 Statistical analysis

The JMP 5.0.1 (SAS Institute) was used to perform statistical analyses using one‐way analysis of variance (ANOVA). The Tukey multiple comparison test was applied to determine if there was a significant difference among the means of the samples using *p* < .05 level of significance.

## 3 RESULTS AND DISCUSSION

### 3.1 Physicochemical and textural properties of vegan‐based cheese formulations

In the beginning, the vegan‐based cream cheese samples produced according to the experimental design were characterized in terms of some basic physicochemical and textural analyses. The pH value of cream cheese is an important parameter for cheese hardness. As the pH increases, the hardness value of the cheese decreases (Monteiro et al., 2009). Therefore, setting the pH of cream cheese is one of the most important steps during the cheese production. The pH values of vegan‐based cream cheese products varied between 4.44 and 4.89. The pH value of commercial cream cheese was measured to be 5.06. It was determined that the pH decreased due to the addition of citrus fiber into the vegan‐based cream cheese formulation. When the pH reaches 4.6 during the cream cheese production process, the caseins form a network and a gel structure if their concentration is large enough (Coutouly et al., 2013). This difference is acceptable as the formation of the cheese structure takes place at pH 4.6. In the study performed by Gigante et al. (2006), they observed the effects of temperature and pH on cream cheese during storage, and cream cheese having a pH of 4.8 was chosen as a control sample. The water activity, which characterizes the moisture binding energy of a product, is important for evaluating the changes in food structure and intermolecular interactions of all added substances (Grek et al., 2022). The water activity values of vegan‐based cream cheese products varied between 0.88 and 0.90, while the water activity value of commercial vegan cheese was measured as 0.88. The water activity value of produced cheese samples at the trial points was found to be very close to that of the commercial cream cheese. The lightness (L*) value of all vegan‐based cream cheese samples ranged from 77.53 to 89.72. The L* value of the commercial cream cheese was found to be 89.72. L* values of the cream cheese samples were closer to each other, but the addition of citrus fibers decreased the lightness values of the samples significantly (*p* < .05) compared to the control. All samples had a negative a* (greenness) value and these values ranged from (−0.40) to (−1.83) for all samples. The commercial cream cheese had the lowest a* value and the closest sample to the control is the cheese sample produced with 15% fat and 1.5% fiber (2). Although there was no linear increase in color values as the amount of citrus fiber increased, a* value was found to be higher for all cheese samples compared to the control. The addition of citrus fiber caused a decrease in b* values of all cheese samples. The lowest b* value was recorded for the sample including 30% fat and 1.5% fiber (9) and the highest b* value was measured in the sample prepared with the addition of 22.5% fat and 1.5% fiber (6).

**FIGURE 2:**
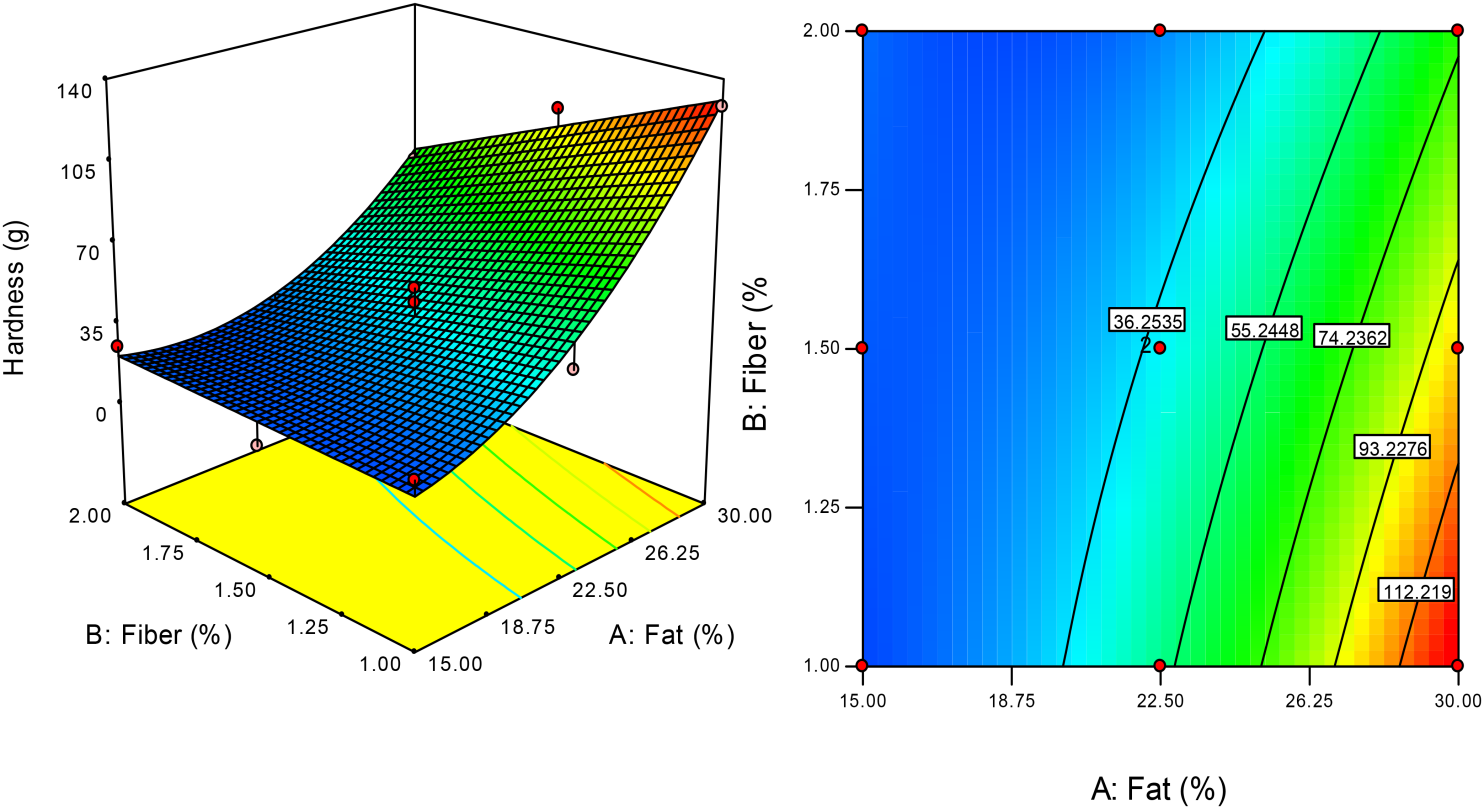
Effect of processing variables on the hardness value of vegan‐based cream cheese samples.

Texture analysis was applied to the commercial cream cheese and the vegan‐based cream cheese samples produced using different ratios of fat and citrus fiber components. The hardness values of the vegan‐based cream cheese samples were significantly different from that of the commercial cream cheese 78.0 (g). The vegan‐based cream cheese containing 15% fat and 1.5% citrus fiber (sample 2) exhibited the lowest hardness values Table 1. Vegan‐based cream cheese sample containing 30% fat showed the highest hardness values among the produced samples. Figure 2 illustrates the change in hardness values of the cheese sample depending on the processing variables. When the samples containing similar amounts of dietary fiber were compared, it was determined that the hardness value of the samples increased as the fat content increased. According to the study of Perina et al. (2015), the vegetable oil emulsion additive and passion fruit peel powder in yogurt had a significant effect on firmness. The firmness is higher in yogurts with vegetable oil and peel powder when it was prepared with skim milk, otherwise a whole milk yogurt with peel powder reduces the firmness of yogurt (Perina et al., 2015).

The effect of processing variables on the hardness parameter, which is the dependent variable (response), was analyzed and the fitted model was subjected to analysis of variance (ANOVA) to specify significance (*p* < .05), coefficient of determination (*R* ^2^), and lack of fit. The fitted model for hardness was significant (*p* < .05), and the lack of fit was not significant as desired (0.8525) (Table 2). The insignificance of the lack of fit suggested that the model was adequate for the fitting (Gharibzahedi et al., 2013). By using Design‐Expert software, the desirability values were calculated. The amount of fat and citrus fiber values required to obtain the hardness value in the desired optimum range (%75–90) was calculated. The formulations selected for the optimum hardness value were A: 26.31%–B: 1.21%, A: 28.81%–B: 1.41%, and A: 29.15%–B: 1.6%.

These optimized values were applied to produce three different vegan‐based cream cheese samples (A, B, and C) with the mixture of other ingredients given in Table 3 and compared to measured properties of commercial vegan‐based cream cheese.

The following quadratic equation of function A: fat and B: citrus fiber was fitted for each factor assessed at each experimental point. The final model equation used was:

$$\text{Hardness}\;\left(g\right)=34.65+41.18\times A-13.93\times B-15.74\times A\times B+25.72\times A^{2}$$

### 3.2 Water activity of the vegan‐based cream cheeses

Water activity results of the samples are shown in Table 4. Water activity values were in the range of 0.87–0.88. The water activities of vegan‐based cream cheese samples and commercial vegan cream cheese were determined to be similar (*p* > .05). Water activity is an important factor for food safety and stability, as it is related to microbial and chemical deterioration in foods (Ostrowska‐Ligęza & Lenart, 2015). According to the study performed by Møller et al. (2012), they determined that the water activity of 11 cream cheese samples ranged between 0.986 and 0.997. In addition, Lemes et al. (2016) reported that the water activity values of cream cheese samples were in the range of 0.962 and 0.958. Comparing water activity of our samples with these reports (Lemes et al., 2016; Møller et al., 2012), it can be concluded that in the current suggested formula, vegan‐based cream cheese samples may have better stability and shelf life.

**TABLE 4:** pH, water activity (*a*_*w*_), L*, a*, and b* results of vegan‐based cream cheeses with citrus fiber.^†^

Sample^‡^	pH	Water activity (*a*_*w*_)	Firmness	L*	a*	b*
A	4.78 ± 0.05^C^	0.88 ± 0.00^A^	24.12 ± 4.44^C^	78.52 ± 0.91^B^	−0.10 ± 0.03^A^	12.89 ± 0.60^A^
B	4.83 ± 0.02^B^	0.88 ± 0.00^A^	87.66 ± 6.33^A^	78.83 ± 0.39^B^	−0.14 ± 0.01^A^	13.07 ± 0.32^A^
C	4.91 ± 0.01^B^	0.88 ± 0.01^A^	68.51 ± 15.04^A^	78.33 ± 1.05^B^	−0.34 ± 0.06^B^	13.39 ± 0.40^A^
D	5.71 ± 0.07^A^	0.87 ± 0.00^A^	42.30 ± 1.72^B^	90.81 ± 0.95^A^	−0.85 ± 0.11^C^	5.56 ± 0.33^B^

^†^ Different superscript letters in the same column show significant difference (*p* < .05).

^‡^ A: sample with 1.21% citrus fiber, B: sample with 1.41% citrus fiber, C: sample with 1.60% citrus fiber, and D: commercial vegan‐based cream cheese.

### 3.3 pH values of the vegan‐based cream cheeses

The pH value is an important factor in determining the texture of cheeses (Grossmann & McClements, 2021). pH values of the vegan‐based cream cheese samples and commercial vegan‐based cream cheese are shown in Table 4. Commercial vegan cream cheese had a higher pH compared to vegan‐based cream cheese samples. Commercial vegan cream cheese contains pea protein, and pea protein gels were formed at pH 5.65 (Sun & Arntfield, 2010). Therefore, the pH value of commercial vegan‐based cream cheese was found to be 5.71. The pH values of the samples were 4.78, 4.83, and 4.91 for A, B, and C, respectively, demonstrating that the increase in citrus fiber level in the formulation significantly affected the pH values of the samples (*p* < .05). Vegan‐based cream cheeses were added with citrus fiber in addition to pea protein. Sayas‐Barberá et al. (2012) reported that there was a decrease in the pH value of traditional sausages enriched with citrus fiber because of increased acidification due to fructose and ribose in the citrus fiber being used as a substrate for the lactic acid bacteria (LAB) population and lactic acid bacteria. Furthermore, Srimali et al. (2019) produced a yogurt drink containing 0.2% citrus fiber as a stabilizer and compared it with samples containing 0.3% gelatin and 0.035% carrageenan, and detected that the highest acidity was determined in yogurt drink added with 0.2% citrus fiber.

### 3.4 Color properties of the vegan‐based cream cheeses

The color properties of the vegan‐based cream cheeses and commercial vegan cream cheeses are given in Table 4. Color parameters are significant for consumer acceptance and commercial value of the product (Zare et al., 2011). The commercial vegan‐based cream cheese differed in lightness and chromaticity, a* and b* values concerning all other vegan‐based cream cheeses (*p* < .05). Differences in fat and fiber in the vegan‐based cream cheese recipe did not cause a difference in the L* value of the products (*p* > .05). Lightness (L*) value was found to be lower in vegan‐based cream cheese containing citrus fiber compared to the commercial product. This situation may be an indication that the addition of citrus fiber increases the opacity of the product. According to the study performed by García‐Pérez et al. (2005), they found that the addition of orange fiber decreased the L* value, and it was thought that the fiber had a darkening effect due to the absorption of water by the fiber. The a* (redness) and b* (yellowness) values of the fiber‐containing vegan cream cheese samples were higher than those of the commercial vegan cream cheese samples. Fernández‐Ginés et al. (2003) found that the Bologna sausage enriched with citrus fiber had higher a* and b* values than those of the control. These color differences could be reasoned by the presence of the carotenes in the structure of citrus fiber.

### 3.5 Textural characteristics of the vegan‐based cream cheeses

The hardness values of the vegan‐based cream cheeses and control sample are displayed in Table 4. The sample produced with the addition of 1.21% citrus fiber (A) showed similar hardness values to the control sample. When the amount of citrus fiber in the sample increased, an increment in the hardness value of the cheese samples increased. Although B and C samples had different amounts of citrus fiber, there was no significant difference in terms of hardness values (*p* > .05). In the study conducted by Hennelly et al. (2006), they compared the hardness values of cheese alternative products added with inulin as a fat substitute and they reported that the hardness value of the samples increased significantly with the addition of inulin (*p* > .05), but there was no significant difference in hardness values of the cheese samples containing different levels of inulin (*p* < .05).

### 3.6 Microstructure properties of vegan‐based cream cheeses

**FIGURE 3:**
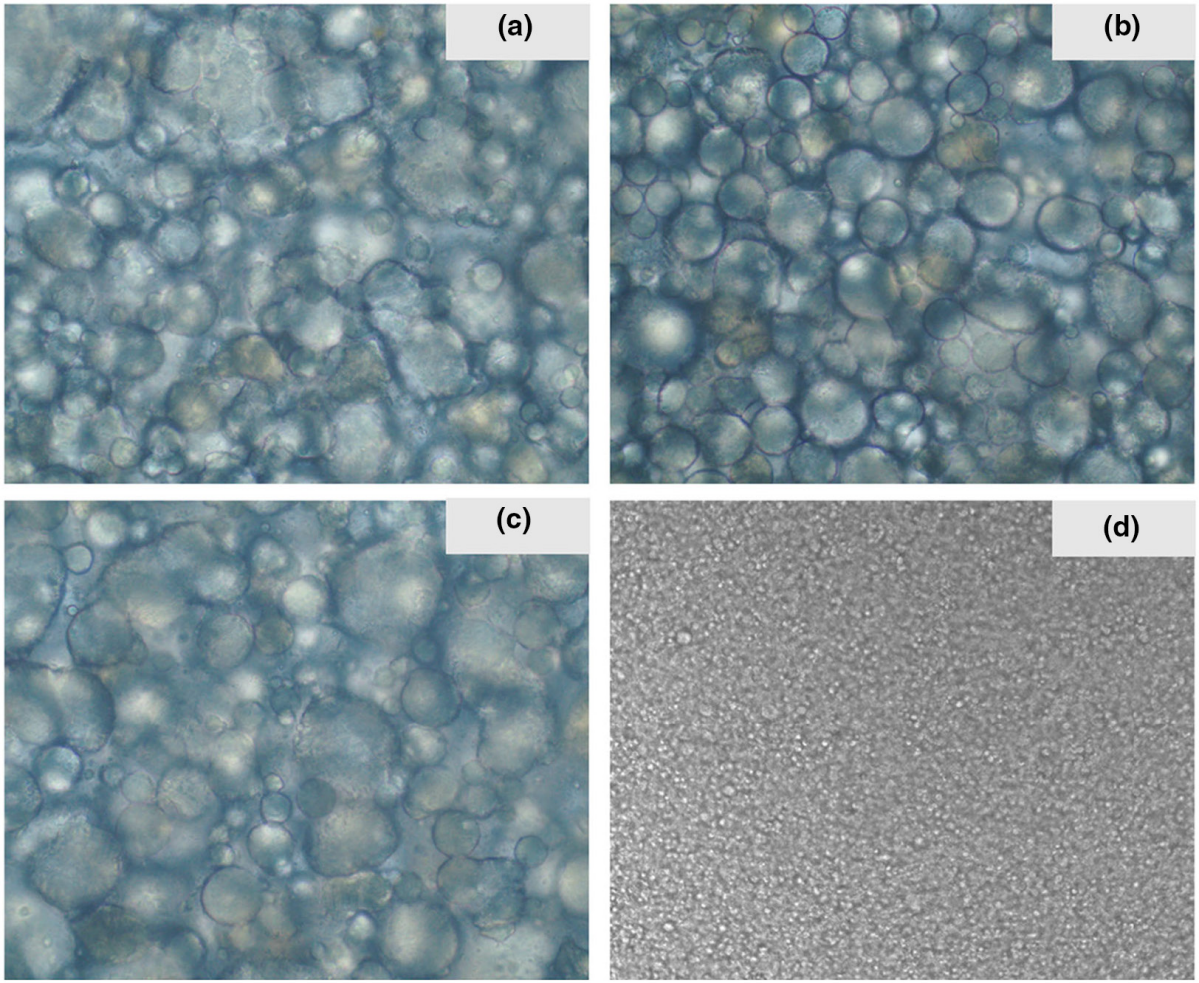
The microstructure of the vegan‐based cream cheese samples by polarized light microscopy with 40× objective. (a) Sample with 1.21% citrus fiber, (b) sample with 1.41% citrus fiber, (c) sample with 1.60% citrus fiber, and (d) commercial vegan‐based cream cheese.

In this study, polarized light microscopy was used to investigate the microstructure of samples and commercial vegan‐based cream cheeses, and the microstructure images of these samples are shown in Figure 3. All vegan‐based cream cheeses showed exactly spherical droplets, which could be reasoned to have been caused by the fat droplets. It is clearly seen that the ring distribution and size were not completely homogeneous in the vegan‐based cream cheeses. However, the rings in the structure of commercial vegan cream cheese were distributed more homogeneously and were of smaller size. The difference in the size of corpuscular structure may be related to the fat aggregates and their variable particles, as well as the pH value of cheese (Ong et al., 2020).

The presence of blur in the image of samples can be associated with the amount of citrus fiber in the formulations. The droplet sizes were typically similar in all samples because there was not any significant difference in the fat amounts of the samples. Wendin et al. (2000) used a confocal laser scanning microscope to image the microstructure of cream cheeses and they reported that the level of fat increased in the formulation, and similar droplet sizes were recorded. In another study, the microstructure of cream cheese was examined using confocal laser scanning microscopy (Ong et al., 2020). At different pH values, different microstructures of cheese samples were observed. The findings recorded fat‐free particles and large corpuscular structures >10 μm in cheese samples. It was concluded that a decrease in the pH value of cheese caused a denser microstructure, smaller corpuscular structure, merged accumulated β‐sheet structure, and a firmer cheese products (Ong et al., 2020).

### 3.7 Sensory evaluation scores of the vegan‐based cream cheeses

The sensory evaluation scores of vegan‐based cream cheeses and commercial vegan‐based cream cheese (sample D) recorded according to eight different sensory parameters are presented in Table 5. Regarding the admiration of vegan‐based cream cheese formulations and commercial vegan‐based cream cheese, there were no differences in appearance, color, taste, and odor qualities (*p* > .05).

**TABLE 5:** Sensory analysis of vegan‐based cream cheeses with citrus fiber^†^.

Sensory parameter	A	B	C	D
Appearance	3.75 ± 0.62^A^	3.83 ± 0.57^A^	3.58 ± 0.90^A^	4.08 ± 0.79^A^
Color	3.91 ± 0.79^A^	4.08 ± 0.79^A^	3.83 ± 0.71^A^	3.83 ± 0.83^A^
Spreadability	4.00 ± 0.42^A^	4.16 ± 0.71^A^	3.33 ± 0.65^B^	4.25 ± 1.05^A^
Texture/mouthfeel	3.08 ± 0.79^B^	3.50 ± 0.67^AB^	3.25 ± 0.86^B^	3.83 ± 1.11^A^
After‐taste	3.08 ± 0.79^B^	3.33 ± 0.77^B^	3.08 ± 0.90^B^	3.83 ± 0.93^A^
Taste	3.33 ± 0.77^A^	3.50 ± 0.90^A^	3.00 ± 1.2^A^	3.41 ± 1.37^A^
Odor	3.41 ± 0.66^A^	3.66 ± 0.77^A^	3.16 ± 1.02^A^	3.83 ± 1.26^A^
Overall acceptance	3.36 ± 0.62^B^	3.66 ± 0.98^A^	2.83 ± 0.83^C^	3.75 ± 1.05^A^

^†^ Different superscript letters in the same line show significant difference (*p* < .05).

The cheese added with the highest amount of fat and citrus fiber showed the lowest spreadability value and the differences among the samples were found to be significant (*p* < .05). The reason for the reduction in the spreadability evaluated by the panelist may be related to the absorption of high moisture by the citrus fiber due to its higher water‐holding capacity. In addition, the crystallization of the fat in the cream cheese may have affected the spreadability. In their study, Brighenti et al. (2008) found that cream cheeses with high fat had lower spreadability than non‐fat cheeses. While the texture/mouthfeel value of the B sample was scored as closest to commercial vegan cheese by the panelists, the texture/mouthfeel value of the sample with the lowest fiber content (A) was scored lowest. A similar result was found within the after‐taste criterion and sample B was scored close to sample D by the panelists. There were no significant differences (*p* > .05) between the B and D samples with respect to overall acceptance. The sample with the highest fiber content (C) was scored lowest for overall acceptance significantly (*p* < .05) by the panelists.

## 4 CONCLUSIONS

In this study, citrus fiber was used to enrich and structure the cream cheese formulation to produce vegan‐based cream cheese. The amount of fat and citrus fiber values was required to be evaluated at the desired level to achieve a hardness near 42.304 (g) for commercial cream cheese. The formulations selected for the optimum hardness value were found with the combination of fat and citrus fiber as: fat: 26.31%–citrus fiber: 1.21%, fat: 28.81%–citrus fiber: 1.41%, and fat: 29.15%–citrus fiber: 1.6%. According to the results, it was observed that the use of citrus fiber in the production of cream cheese was acceptable. Textural characteristics and sensory properties of the vegan‐based cream cheese samples were similar to those of the commercial cream cheese and these vegan‐based cream cheeses have the potential to be a promising option for the cream cheese. As a result, citrus fiber has shown great promise as a filling component in non‐dairy products.

## AUTHOR CONTRIBUTIONS

**Basak Gurbuz:** Data curation (equal); formal analysis (equal); investigation (equal); methodology (equal); resources (equal); writing – original draft (equal). **Merve Cayir:** Data curation (equal); investigation (equal); methodology (equal); writing – original draft (equal); writing – review and editing (equal). **Esra Akdeniz:** Formal analysis (equal); investigation (equal); methodology (equal); writing – original draft (equal). **Saniye Akyıl Öztürk:** Data curation (equal); writing – original draft (equal). **Safa Karaman:** Resources (equal); writing – original draft (equal). **Atefeh Karimidastjerd:** Writing – review and editing (supporting). **Omer Said Toker:** Investigation (equal); resources (equal); supervision (equal); writing – original draft (equal); writing – review and editing (equal). **Nevzat Konar:** Writing – review and editing (supporting).

## CONFLICT OF INTEREST STATEMENT

All authors declare no conflicts of interest.

## Data Availability

Research data are available within the article.
